# Electrical Microenvironment Reconstruction and the Application of Biomaterials in Spinal Cord Injury

**DOI:** 10.3390/jfb17040172

**Published:** 2026-04-01

**Authors:** Jie Zhang, Xiangyun Zou, Mengshuang Li, Yaosai Liu

**Affiliations:** 1 Department of Neurosurgery, Qilu Hospital (Qingdao), Cheeloo College of Medicine, Shandong University, 758 Hefei Road, Qingdao 266035, China; 2 Department of Neurosurgery, Beijing Tsinghua Changgung Hospital, School of Clinical Medicine, Tsinghua Medicine, Tsinghua University, Beijing 100084, China; 3 Department of Pediatrics, Qilu Hospital (Qingdao), Cheeloo College of Medicine, Shandong University, 758 Hefei Road, Qingdao 266035, China; 4 Department of Radiology, Qilu Hospital (Qingdao), Cheeloo College of Medicine, Shandong University, 758 Hefei Road, Qingdao 266035, China

**Keywords:** spinal cord injury, bioelectric microenvironment restoration, conductive biomaterials, electrical stimulation

## Abstract

The key challenge in restoring neural function after spinal cord injury stems from a vicious cycle triggered by the collapse of the bioelectrical microenvironment at the injury site: an ‘electrical silence–neuronal degeneration–glial proliferation’ cascade that conventional therapies fail to reverse. This review systematically summarizes the pathological mechanisms of electrical microenvironment imbalance and its critical role in neural regeneration. Furthermore, current intervention strategies based on biomaterials are outlined: evolving from passive reconstruction of electrical pathways using conductive materials to proactive regulation of local electric fields through exogenous electrical stimulation, which activates key signaling pathways, such as voltage-gated calcium channels, and thereby promotes axonal regeneration, stem cell differentiation, and immune modulation. Although existing strategies face challenges in precision and biocompatibility, this review integrates multidisciplinary perspectives from neuroscience and biomaterials to establish a theoretical framework for designing precise, biocompatible electrically modulating biomaterials. Ultimately, we aim to advance spinal cord injury treatment from local electrical environment restoration toward a paradigm shift toward functional neural circuit reconstruction.

## 1. Introduction

Spinal cord injury is a common type of central nervous system injury [1]. The spinal cord, which together with the brain forms the central nervous system, acts as the essential conduit for both descending motor signals from the brain to the peripheral nerves and ascending sensory signals from the peripheral nerves to the brain. Caused by primary injury, including trauma, tumors, inflammation, and other factors, spinal cord injury often disrupts these ascending and descending signaling pathways, leading to severe neurological dysfunction and has become one of the primary causes of disability worldwide [1]. Although neural tissue exhibits limited endogenous repair capacity, the regenerative potential of the central nervous system in adults is extremely restricted [2]. Additionally, spinal cord injury is commonly accompanied by secondary injury, including regional vascular disruption, persistent inflammatory responses, and subsequent edema, which further exacerbate neurological damage [2]. During the subacute to chronic repair phase, multiple inhibitory microenvironmental factors, including ongoing damage from inflammation and glial scar proliferation, impede neural regeneration, making post-injury repair challenging [3]. Traditional treatment strategies (such as surgical decompression, anti-inflammatory therapy, and dehydration therapy) primarily focus on early symptomatic management to minimize injury, lacking effective approaches for neural reconnection and functional recovery [4,5]. Although methods like rehabilitation therapy and ganglioside administration hold promise for promoting neuroregeneration, their efficacy remains limited [6].

Against this backdrop, biomaterials are gaining increasing attention in the field of neural regeneration following spinal cord injury. Among these, biomaterials that modulate the bioelectrical microenvironment, a key physical cue playing a central regulatory role in neural development, axon guidance, and injury response, are emerging as a critical target for overcoming bottlenecks in neural regeneration.

As essential functional units within the bioelectrical signaling network, neurons form a highly interconnected electrophysiological network through the generation and conduction of action potentials, precisely regulating the body’s motor, sensory, and autonomic functions. This network not only facilitates information transmission but also fundamentally maintains neuronal viability, metabolic homeostasis, and synaptic plasticity [7]. Prolonged absence of electrical activity can trigger neuronal dedifferentiation, axonal degeneration, and even programmed cell death [8]. Within central nervous system tissues, such as the spinal cord, endogenous electrical activity is crucial for maintaining the excitatory-inhibitory balance of neural networks [9]. Injury-induced disruption of electrical conduction not only causes signal transmission failure but also induces localized electrical silence, accelerating neuronal functional decline and glialization [10,11]. This creates a vicious cycle that impedes regeneration. Therefore, restoring and sustaining electrophysiological homeostasis in the injured region has become a core objective of neuroregeneration strategies.

To address this challenge, the paradigm of neural regeneration is undergoing a profound shift from passive structural support to proactive electrophysiological regulation. On one hand, conductive biomaterials mimic the electrical properties of natural neural tissue, establishing continuous electrical bridges at injury sites to restore local electrical conduction pathways and provide electrophysiological guidance for axonal regeneration. On the other hand, exogenous electrical stimulation techniques directly activate the electrical activity of surviving neurons by applying controllable electrical signals, reversing electrically silent states, and initiating regeneration-related molecular programs [12]. The synergistic action of these 2 approaches collectively aims to reconstruct a functionally active electrical microenvironment within the injured region. This approach transcends the traditional role of biomaterials as mere physical supports, advancing toward the functional reconnection of neural circuits [13].

**Figure 1:**
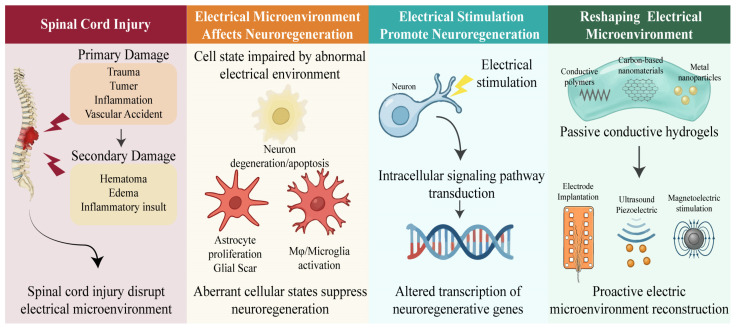
Overview of electrical microenvironment and reconstruction strategies following spinal cord injury. (**Left panel**): Primary injuries trigger secondary damages, including hematoma, edema, and inflammatory insult, which disrupt the spinal cord’s native electrical microenvironment. (**Center left panel**): This aberrant electrical microenvironment impedes neuroregeneration by inducing neuron apoptosis, astrocyte proliferation, and Mφ/microglia activation. (**Center right panel**): Electrical stimulation activates intracellular signaling pathways, inducing altered transcription of neuroregenerative genes. (**Right panel**): Electrical microenvironment reconstruction shifts from passive conductive hydrogels to proactive strategies, including electrode implantation, piezoelectric, and magnetoelectric stimulation.

This review systematically outlines cutting-edge advances in bioelectric-integrated biomaterials for neural regeneration, covering (1) the physiological basis of the bioelectrical microenvironment and its pathological evolution post-injury; (2) cellular and molecular mechanisms by which electrical stimulation regulates neuronal fate and axonal regeneration; (3) passive reconstruction of bioelectric signaling environments using conductive biomaterials; and (4) proactive bioelectric microenvironment reconstruction, ranging from electrode implantation to in situ electrically stimulated nanoparticles (Figure 1). By integrating perspectives from neuroscience, biomaterials science, and bioelectronic engineering, this paper elucidates how electrically regulated strategies advance from microenvironmental remodeling to functional neural network reconstruction. It provides a theoretical framework and technical roadmap for designing and clinically translating next-generation materials for post-spinal cord injury neural regeneration.

## 2. Bioelectric Microenvironment Imbalance: The Core Problem in Neuroregenerative Barrier Formation Following Spinal Cord Injury

Following spinal cord injury, the collapse of the bioelectric microenvironment constitutes the core pathological mechanism underlying regenerative barriers. Primary mechanical injury compresses local neuronal axons, leading to massive neuronal death and axonal rupture [14]. Furthermore, subsequent ischemia and hypoxia exacerbate damage to surviving neurons [15]. Hematomas and edema further intensify local compression on neurons following the primary injury [16,17]. The combined effects of these factors directly cause complete interruption of electrical conduction in the core lesion area. Although severed axon terminals are rapidly sealed by local cell membranes post-injury, the loss of ion channels on the cell membrane surface and the irreversible impairment of exocytosis-mediated neurotransmitter release at axon terminals prevent the timely restoration of normal neural signaling and energy supply. Secondary cascading reactions triggered by ischemia-hypoxia and inflammation (such as excitotoxicity and oxidative stress) further expand the area of electrical silence. This dual mechanism causes surviving neurons to experience calcium overload and uncontrolled opening of sodium-potassium channels, leading to loss of action potential function [18]. Simultaneously, the absence of electrical input triggers apoptosis in these surviving neurons. Beyond the altered electrical responsiveness caused by the neurons’ own morphological defects, the glial scar formed by proliferating reactive astrocytes not only creates a physical barrier. Its high-density glial fibers and increased deposition of chondroitin sulfate proteoglycans (CSPGs) significantly raise local electrical resistivity, forming an ‘electrical insulation barrier’ that blocks the electrical coupling of surviving axons across the injury site [19]. As a crucial component of the central nervous system and a key element of myelin sheaths in the normal physiological environment, oligodendrocytes are influenced by the electrical environment. They can exchange chemical energy with neurons, thereby promoting the maintenance of normal physiological neuronal activity. During impulse transmission, calcium influx and potassium efflux triggered by action potentials activate potassium channels (Kir4.1) on oligodendrocytes and downstream calcium signaling pathways [20]. This promotes upregulation of energy metabolism within oligodendrocytes, enabling them to supply neurons via the lactate shuttle mechanism [20]. However, in the absence of electrical signals, this lactate shuttle mechanism between oligodendrocytes and neurons fails to function. Consequently, oligodendrocytes contribute less to maintaining neuronal homeostasis, making it increasingly difficult to sustain neuronal stability. Macrophages, also influenced by electrical signals, participate in modulating neuronal ion channels under physiological conditions [21]. Their own membrane potential affects their activation process [22]. In the absence of an electrical network, macrophage activation is promoted, further increasing inflammatory levels. This secondary, multi-cellular electrically inhibited environment following primary injury results in significantly reduced motor evoked potential (MEP) amplitudes below the injury level even in patients with non-completely severed spinal cord injuries [23]. This indicates that even anatomically intact nerve fibers lose their electrical conduction function due to microenvironmental instability. This vicious cycle of ‘electrical silence-neuronal degeneration-glia proliferation’ plunges the injured region into electrophysiological dysregulation. Traditional intervention strategies targeting only biochemical factors struggle to reverse this pathological state, necessitating the reconstruction of electrical signaling.

## 3. Mechanisms of Electrical Signals in Promoting Neural Regeneration

**Figure 2:**
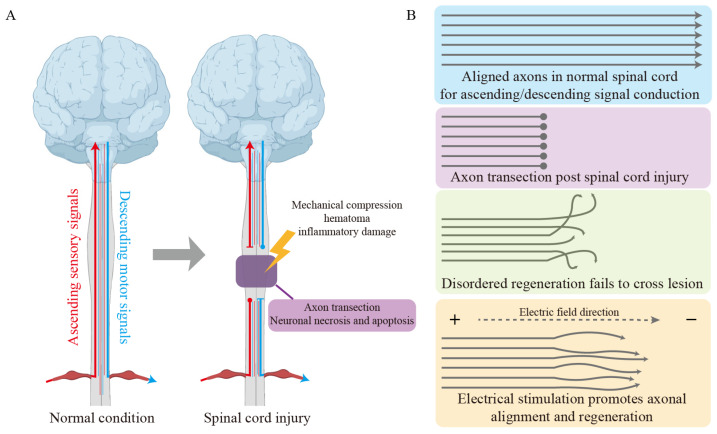
Schematic diagram of spinal cord injury and electrical stimulation-promoted regeneration. (**A**) Descending motor and ascending sensory signal transmission under normal conditions and pathway interruption after spinal cord injury. (**B**) Aligned axons in the normal spinal cord enable ascending and descending signal conduction, but following injury, axons are transected, and regeneration becomes disordered, failing to cross the lesion. Electrical stimulation effectively promotes axonal alignment and regeneration.

Electrical signals exert multi-level, multi-target regulatory effects during neural regeneration. The migration of endogenous neural stem cells and neurons toward the injury site constitutes the initial step of neural regeneration. Electrical stimulation can promote the directed migration of endogenous neural stem cells and neurons, a phenomenon termed electroguidance [24]. Studies on dorsal root ganglion (DRG) neurons from chicken embryos have demonstrated that applied electric fields effectively guide cell migration [25]. Similarly, rat neural stem cells (NSCs) successfully migrated from the subventricular zone (SVZ) to the striatum following stroke under electrical stimulation, demonstrating the ability of electrical stimulation to guide cell migration across species in neuronal and neural stem cell models [26]. Notably, electrical stimulation significantly enhances the differentiation and proliferation of neural stem cells, potentially mediated through the Wnt signaling pathway [27,28,29]. Furthermore, electrical stimulation promotes neuronal alignment, enabling neurons to form highly organized neural networks aligned along the electric field direction from the anode to the cathode (Figure 2) [30,31]. This alignment is crucial for reconstructing ascending and descending neural connections. Electrical stimulation also exerts significant regulatory effects on immune cells.

**Figure 3:**
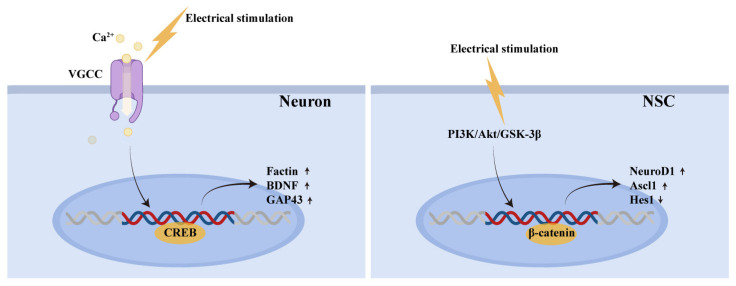
Key gene-level changes following electrical stimulation. (**left panel**) In neurons, electrical stimulation triggers Ca^2+^ influx through VGCCs, activating the transcription factor CREB, which upregulates neuroregeneration-associated genes such as BDNF and GAP43. (**right panel**) In NSCs, electrical stimulation activates the PI3K/Akt/GSK-3β signaling pathway, upregulating proneural factors (NeuroD1, Ascl1) and downregulating the Notch effector Hes1 via β-catenin nuclear translocation.

The core mechanism underlying these changes lies in the electrical signal’s ability to remodel the local bioelectric microenvironment. This activation triggers voltage-gated ion channels on the cell membrane, particularly voltage-gated calcium channels (VGCCs), facilitating extracellular Ca^2+^ influx and thereby initiating a cascade of signal transduction events (Figure 3).

At the neuronal level, electrically induced membrane depolarization prompts VGCC conformational changes, causing transient intracellular Ca^2+^ concentration spikes. This calcium signal not only directly carries electrical activity but also serves as a key second messenger to activate calcium/calmodulin-dependent protein kinase II (CaMKII) [7,32]. It synergistically stimulates adenylate cyclase (AC) activity, significantly elevating cyclic adenosine monophosphate (cAMP) levels. Subsequently, cAMP-dependent protein kinase A (PKA) phosphorylates the transcription factor CREB, initiating the transcriptional expression of regeneration-related genes such as GAP-43 and BDNF [33,34]. Furthermore, the Ca^2+^-CaMKII signaling pathway activates the dual leucine zipper kinase (DLK/MAP3K12) pathway, promoting phosphorylation of microtubule-associated proteins and cytoskeletal reorganization [35]. Concurrently, CREB influences growth cone morphoplasticity by regulating F-actin dynamics, collectively driving axonal extension [36]. Crucially, applied electric fields induce a spatially asymmetric distribution of growth cone membrane potentials, activating Rac1/Cdc42 GTPases [37]. This promotes preferential actin filament polymerization on the cathode side and microtubule extension along the electric field vector, enabling electrotropic axonal guidance [37].

For endogenous or transplanted NSCs, electrical signals not only enhance proliferation but also significantly promote directed differentiation toward the neuronal lineage [38,39]. This process involves activation of the PI3K/Akt/GSK-3β/β-catenin signaling axis, stabilizing β-catenin nuclear translocation to drive neuron-specific gene expression like NeuroD1 [40]. Additionally, electrical signals upregulate Ascl1 expression and promote sustained accumulation of its products while simultaneously downregulating Hes1, thereby lifting transcriptional suppression on neuronal differentiation and reinforcing neuronal fate determination [41].

Furthermore, electrical signals can modulate the local immune microenvironment. Studies indicate that electrical stimulation promotes the directed migration of microglia [42]. In bone and skin regeneration models, electrical stimulation has been shown to induce polarization from the pro-inflammatory M1 phenotype to the anti-inflammatory, reparative M2 phenotype [43,44,45]. Although evidence within the central nervous system requires further validation, this immunomodulatory effect holds promise for mitigating secondary inflammatory injury and creating a more favorable microenvironment for neural regeneration.

By synergistically regulating intrinsic neuronal regeneration programs, stem cell fate determination, and local immune responses, electrical signals constitute a key intervention target for functional recovery after spinal cord injury. Constructing controllable electrical signal microenvironments using biomaterials holds promise as an innovative therapeutic strategy to promote spinal cord injury repair.

## 4. Conductive Biomaterials Passively Reconstruct the Bioelectric Signaling Environment

By mimicking the electrical properties of natural neural tissue, conductive biomaterials provide continuous electronic pathways to injured areas. They passively utilize endogenous electrical signals from residual neurons to restore the local electrical environment. Existing hydrogels for spinal cord regeneration are mainly composed of natural polymers such as gelatin, agarose, and hyaluronic acid, which offer excellent biocompatibility and mimic the native extracellular matrix. Although the water-containing properties of hydrogel materials inherently confer some conductivity, research has increasingly explored ways to reduce electrical resistance to more effectively minimize obstacles to electrical signal transmission. To this end, various conductive materials have been integrated into hydrogel systems to enhance conductivity (Table 1). Commonly used conductive materials include metals, polymers, and other materials derived from both natural and synthetic sources. These conductive fillers encompass a wide range of materials such as conductive polymers, carbon-based nanomaterials, metallic nanomaterials, and phosphorus-based nanomaterials.

### 4.1. Conductive Polymers

Conductive polymers have emerged as a mainstream choice due to their tunable electrical properties and excellent biocompatibility. Polypyrrole (PPy) can be chemically grafted and integrated into hydrogel networks [61]. While offering good biocompatibility and being widely used in spinal cord injury treatment research, its electrical conductivity is relatively poor [61]. Polyaniline (PANI) exhibits superior stability but poor biocompatibility [62]. It has been shown to promote local macrophage apoptosis via ROS and MMP formation mediated by the caspase-3 pathway, raising concerns about its biological safety [63]. Poly(3,4-polyvinylpyridine) (PEDOT) combines the advantages of both, offering excellent conductivity, good biocompatibility, and stability [64]. Although degradation in biological fluid environments may affect its electrochemical properties, it remains a significant conductive polymer choice [65,66]. Conversely, other research indicates that it may exert immunotoxicity on macrophages via the NF-κB signaling pathway and oxidative stress [67]. Current applications of conductive polymers in spinal cord injury include gelatin/PPy injectable hydrogels, which significantly promote NSC migration and exhibit favorable therapeutic outcomes [47]. Hydrogels combining PPy with polyphenols/tannic acid, as well as those incorporating agarose/gelatin, have also demonstrated promising therapeutic effects [46,48]. Through grafting onto gelatin, PANI has also demonstrated favorable therapeutic outcomes in spinal cord injury treatment [49]. PEDOT exhibits promising therapeutic effects in spinal cord injury treatment, whether used as monomer nanoparticles, doped with chondroitin sulfate methacrylate and tannic acid, or doped with sulfated lignin [50,51,52].

### 4.2. Carbon-Based Nanomaterials

Carbon-based nanomaterials leverage their high specific surface area and superior mechanical properties to construct three-dimensional conductive networks. Carbon nanotubes (CNTs) exhibit exceptional conductivity, and their nanoscale topological structure can mimic the orientation of nerve fiber bundles [68]. However, their synthesis process is complex; they are prone to agglomeration, and their cost is high [69]. The relatively poor biocompatibility of CNTs warrants particular attention in spinal cord injury applications. Although CNTs can be excreted via urine, their poor biodegradability poses risks of long-term accumulation within the body without elimination [70]. CNTs can activate NLRP3 inflammasomes similarly to asbestos and even induce pulmonary fibrosis, representing a significant constraint for biological applications [71,72]. Graphene and reduced graphene oxide (rGO) form continuous conductive pathways via π-π stacking. They can be covalently grafted into hydrogel networks, exhibiting excellent conductivity [73,74]. In vivo, they can achieve biodegradation with the assistance of enzymes such as MPO [75]. However, they suffer from poor dispersion and agglomeration issues [73,74]. Evidence also suggests that larger-sized graphene poses more severe biosafety concerns [76]. In vitro, hydrogels prepared by covalently grafting CNTs onto oligo(poly(ethylene glycol)fumarate) (OPF) enhanced PC12 cell adhesion, proliferation, and neuronal differentiation [53]. In vivo, electrospun CNT/GelMA hydrogels have been shown to significantly promote cell proliferation and aligned adhesion when combined with exogenous electrical stimulation, without demonstrating significant biosafety concerns [54]. Graphene and rGO remain underutilized in spinal cord injury treatment. While rGO foams have shown therapeutic efficacy for spinal cord injury, this application has amplified concerns regarding their potential biosafety risks [55].

### 4.3. Metal Nanoparticles

Metal nanoparticles used in conductive hydrogels offer distinct advantages in electrical conductivity. Commonly employed metal nanoparticles include gold nanoparticles and silver nanoparticles. Despite their excellent conductivity, both exhibit poor dispersion and a tendency to agglomerate [77,78]. Gold nanoparticles can be degraded by macrophages and excreted through the kidneys and liver [79,80]. Although silver nanoparticles exhibit properties and effects similar to gold, silver nanoparticles spontaneously oxidize into silver ions in aqueous and oxygen-containing environments [81]. The release of silver ions endows silver nanoparticles with significant antibacterial effects [82]. Silver ions exceeding safe thresholds can cause liver and kidney inflammation [83]. Reports indicate that 28 days after implantation, varying degrees of silver accumulation were observed in the liver, kidneys, and brain [84]. Gold nanoparticles are widely applied in CNS injury [85]. Both gold nanorod-containing and gold nanosphere-containing hydrogels have demonstrated excellent neuroregenerative effects [56,57]. Silver nanoparticles, owing to their immunomodulatory effects, are also employed for regulating microglia activity following spinal cord injury, significantly enhancing neural regeneration [58].

### 4.4. Other Conductive Materials

Black phosphorus nanoparticles are also utilized in the construction of conductive biomaterials. Possessing excellent biodegradability and relatively superior conductivity, black phosphorus nanoparticles are currently a popular choice for conductive biomaterials [86]. However, further exploration is needed to optimize their biodegradability. Although black phosphorus nanoparticles have been demonstrated to degrade into non-toxic phosphates in water, reports indicate they may cause DNA damage, warranting further investigation [87,88]. MXene is also employed in constructing conductive hydrogels, exhibiting good biocompatibility and degradability, but it is prone to oxidation [89,90]. Black phosphorus quantum dots, when combined with epigallocatechin-3-gallate to form hydrogels, have been shown to promote neuronal re-entry into the cell cycle via the Akt-GSK3 pathway, thereby facilitating regeneration [59]. GelMA–MXene hydrogels have also been shown to effectively promote adhesion, directed proliferation, and differentiation of NSCs following spinal cord injury [60].

Despite the wide array of materials available to enhance hydrogel conductivity, it is evident that increasing conductivity alone cannot fully reconstruct the extracellular electrical environment following spinal cord injury. Consequently, many studies combine conductive hydrogels with electrodes for exogenous active electrical stimulation to restore this environment. This conceptual shift profoundly reveals the limitations of relying solely on the inherent conductivity of materials to “passively bridge” residual neural electrical signals, while also highlighting the significant potential of introducing exogenous energy to achieve “active regulation” of the electrical microenvironment. Compared with passive conduction, active electrical stimulation can not only deliver a stronger electric field but, in some techniques, also enable precise control over stimulation frequency and intensity. This allows for more effective activation of VGCCs, as well as guidance of cell migration and axonal directional growth. Based on this, various strategies for proactive reconstruction of the bioelectrical microenvironment have emerged, ranging from traditional implantable electrodes to novel wireless electric stimulation, which opens new avenues for advancing spinal cord injury repair.

## 5. Proactive Bioelectrical Microenvironment Reconstruction: From Electrode Implantation to In Situ Electrostimulation Nanoparticles

Conductive hydrogels that passively utilize residual endogenous electrical signals from neurons can partially restore the local tissue’s conductive environment. However, they remain constrained by the field strength of endogenous electrical signals, consistently failing to achieve satisfactory therapeutic outcomes. The underlying reason is that processes crucial for nerve regeneration—migration, axon outgrowth, and neuronal alignment—all rely on field-dependent mechanisms functioning within physiological voltage ranges [91]. This necessitates shifting bioelectric microenvironment reconstruction from passive conductivity to proactive electrical stimulation. Existing stimulation methods encompass electrode implantation, piezoelectric, magnetoelectric, and inductive bioelectric nanomaterials, each with distinct advantages and limitations.

### 5.1. Electrode Implantation

Electrode implantation represents one of the earliest proactive electrical stimulation approaches applied in clinical practice. Traditional platinum, iridium oxide, and platinum–iridium alloy electrodes can deliver precisely parameterized electrical stimulation via an external power source. However, the modulus mismatch between conventional rigid electrodes and soft tissues leads to persistent mechanical stress after chronic implantation, triggering glial proliferation and increased impedance at the electrode–tissue interface [92]. Bendable electrodes incorporate flexible polymer substrates like polyimide, polydimethylsiloxane, or parylene to minimize mechanical stress at contact points, thereby reducing inflammatory responses and significantly enhancing biocompatibility [93,94,95]. Nevertheless, concerns persist regarding infection and inflammation risks associated with long-term implanted electrodes, particularly due to bio-safety concerns posed by lead wires required for external power input. This prompts a shift in current research toward wireless electrical stimulation.

### 5.2. Ultrasound Piezoelectric Materials

Ultrasound possesses moderate tissue penetration capabilities and spatial focusing precision, making it a significant energy carrier for neural electrical signal modulation. Common piezoelectric materials include organic polymers such as poly-L-lactic acid (PLLA) and polyvinylidene fluoride (PVDF), alongside inorganic piezoelectric ceramics like potassium sodium niobate (KNN), BaTiO_3_, and zinc oxide (ZnO). Piezoelectric nanomaterials generate surface charges under ultrasonic mechanical vibrations, enabling in situ “acoustic-to-electric” conversion. Although studies have developed PLLA and PVDF into spinal cord scaffolds via electrospinning, mechanical mismatch remains a significant limitation for central nervous system applications, despite their broad applications in peripheral tissues [96,97,98,99]. In contrast, piezoelectric ceramic nanoparticles like BaTiO_3_-based particles have been used in spinal cord injury treatment with promising outcomes [100]. However, the chemical stability of BaTiO_3_ hinders degradation, posing a major obstacle to its current application [101]. ZnO is another commonly used nanoparticle for ultrasound-assisted piezoelectric neuroregeneration in spinal cord injury treatment, significantly promoting neural stem cell differentiation [102]. However, concerns about its biosafety have drawn considerable attention, with reports indicating potential multi-organ damage [103]. Among these, glomerular edema induced by ZnO is most pronounced [104]. Additionally, studies report possible central nervous system damage [105]. Compared to BaTiO_3_, KNN exhibits excellent biodegradability while maintaining comparable piezoelectric effects, enabling effective therapeutic outcomes under ultrasound stimulation [106,107,108]. Mechanistically, ultrasonic piezoelectric stimulation synergistically enhances neuronal regeneration by activating both the Piezo1 mechanosensitive channel and the L-VGCC pathway under electrical stimulation [108,109]. However, energy loss and mechanical damage caused by ultrasound as it traverses soft tissues and the lamina impede the efficacy of this approach in deep spinal cord tissues, necessitating its cautious application [110].

### 5.3. Magnetoelectric Materials

The limited penetration of ultrasound through soft tissue and bone barriers has redirected recent research toward magnetic field conversion materials. Magnetoelectric materials (mainly shell–core structured magnetoelectric nanoparticles) feature core magnetostrictive materials such as CoFe_2_O_4_, Fe_3_O_4_, and NiFe_2_O_4_, while the shell typically utilizes materials like BaTiO_3_ and BiFeO_3_. Under externally applied alternating magnetic fields, the magnetostrictive–piezoelectric coupling effect generates surface electric fields, enabling wireless electrical stimulation. Its unique advantage is depth independence: magnetic fields penetrate tissue with minimal attenuation, allowing precise activation of deep spinal neurons while avoiding excessive stimulation of superficial tissues. Existing magnetoelectric nanoparticles applied in the spinal cord, such as BaTiO_3_@Fe_3_O_4_, have demonstrated promising therapeutic outcomes [111,112]. Key safety considerations for existing magnetoelectric nanoparticles extend beyond the aforementioned piezoelectric materials to the core magnetostrictive components. Cobalt ferrite, for instance, may release cobalt ions, potentially causing ROS-mediated cellular damage [113,114]. Although Fe_3_O_4_ nanoparticles are considered relatively safer and have FDA approval for medical applications like imaging due to their chemical stability, the potential local release of iron ions could cause iron-induced necrosis in the central nervous system [115,116]. This poses a significant challenge for the regeneration of fragile nerve stumps after injury and warrants special attention. On the other hand, if not rapidly released, these Fe_3_O_4_ particles can undergo endocytosis by macrophages and be metabolized and excreted through the kidneys [117]. Excess iron ions within physiological ranges can also bind to ferritin for storage in hepatocytes or be utilized in hematopoiesis [118].

Additionally, stimulation methods such as magnetoelectric induction, which converts magnetic fields into electric fields via materials like rGO, GeP_3_, and gold nanowires, have demonstrated efficacy, although conversion efficiency remains limited [119,120,121]. Biofuel cells can directly convert biochemical energy into bioelectric energy by locally converting the metabolic substrate glucose into gluconolactone [122]. However, this process relies on oxygen supply, and the efficiency of electrical energy delivery requires further validation. While currently applied in peripheral nerve conduits, their implementation in the spinal cord remains a significant challenge and requires further breakthroughs [123].

## 6. Conclusions and Prospects

Functional impairment following spinal cord injury fundamentally stems from a systemic collapse of the bioelectrical microenvironment, neuronal death, and axonal disruption that interrupt electrical conduction; secondary cascading reactions expand the electrical silence zone, and the high-impedance barrier formed by glial scarring further blocks electrical coupling among surviving neurons, creating a vicious cycle of ‘electrical silence–neuronal degeneration–glia proliferation’. This pathological understanding has propelled neuroregeneration strategies from traditional biochemical interventions toward electrophysiological microenvironment reconstruction, undergoing a paradigm shift from passive electrical conduction support to proactive electrical stimulation regulation. By simulating the electrical properties of neural tissue to construct ‘electrical bridges’, passive conduction of residual endogenous electrical signals provides foundational electrophysiological guidance for axonal regeneration. However, endogenous signal field strength is insufficient and spatially uneven, making it difficult to effectively activate regenerative programs. Thus, proactive electrical stimulation techniques emerged, ranging from precise spatiotemporal control via flexible implantable electrodes to wireless strategies like piezoelectric ultrasound, magnetoelectric conversion, and bioelectric induction. These methods generate controllable electric fields at injury sites through in situ energy conversion, reversing electrically silent states while synergistically regulating neuronal survival, axonal electrotropic guidance, stem cell-directed differentiation, and immune microenvironment remodeling.

Despite increasingly diverse technical approaches, three core challenges persist. First, there is a mismatch between stimulation precision and tissue heterogeneity: homogeneous electric fields struggle to mimic gray-white matter conductivity gradients and cell-type-specific electrical responses. Second, there is a trade-off between energy conversion efficiency and safety; piezoelectric/ferroelectric materials generally exhibit low conversion efficiency and high energy input risks thermal injury, and the degradation kinetics of biodegradable materials are difficult to precisely align with the regeneration cycle. Third, there is a lack of physiological feedback in stimulation; thus, existing strategies cannot dynamically adjust in real time based on neural activity, and research on whether stimulated neurons can generate corresponding regenerative responses remains insufficient.

Future research should focus on multifaceted reconstruction of the electrical environment. First, more efficient, biocompatible, and minimally invasive electrical stimulation methods must be developed. These methods must take into account not only electrical performance but also stability under dynamic physiological conditions, including resistance to corrosion caused by immersion in cerebrospinal fluid within the spinal canal, as well as the mechanical properties of hydrogels and their compatibility with the spinal cord. Second, the relationship between electrical stimulation and cellular responses must be investigated, together with methods for dynamically adjusting stimulation to match different stages of the regeneration process. In addition, the complex physiological structure of the spinal cord necessitates spatial matching of electrical stimulation intensity and frequency. Whether substructures within the spinal cord can be better regulated for regeneration through different stimulation methods remains to be further explored. Only through the deep integration of biomaterial design, electrophysiological mechanisms, and clinical translation capabilities can we develop therapeutic strategies with genuine clinical application value. Building on this foundation, employing more precise regulatory approaches, such as optimized frequency and intensity to guide neurons across the injured area and reconnect with appropriate target neurons, holds the potential to restore the original motor and sensory neural pathways more effectively, thereby achieving accurate functional repair. This will ultimately enable the achievement of substantial recovery of motor and sensory functions after spinal cord injury and pave the way for clinical application.

## Figures and Tables

**Table 1:** Different Conductive Hydrogels for Spinal Cord Injury Treatment.

Category	Conductive Materials	Hydrogel Composition	Signal Pathways Involved	Reference
Conductive polymers	PPy	agarose/gelatin/PPy	CREB-BDNF	[46]
		collagen/PPy	β-tubulin III upregulation	[47]
		polyphenol/tannic acid/PPy	β-tubulin III upregulation	[48]
	PANI	sodium hyaluronate oxide/gelatine-g-PANI	β-tubulin III upregulation	[49]
	PEDOT	gelatin/hyaluronic acid/PEDOT	Limit astrocyte activation through CD44 receptors	[50]
		gelatin methacrylate/hyaluronic acid methacrylate/PEDOT: sulfonated lignin	Not mentioned	[51]
		GelMA/PEGDA/PEDOT:chondroitin sulfate methacrylate/tannic acid	Not mentioned	[52]
Carbon-based nanomaterials	CNT	oligo(poly(ethylene glycol)fumarate)-CNT-poly(ethylene glycol)-acrylate	F-actin promotes adhesion	[53]
		CNT/GelMA	Not mentioned	[54]
	rGO	rGO	Not mentioned	[55]
Metal nanoparticles	Gold	glycol chitosan-oxidized hyaluronate/gold nanosphere-ursodeoxycholic acid	Inhibit inflammatory signals by the MAPK signal pathway	[56]
		hyaluronic acid/gelatin/gold nanorod	Not mentioned	[57]
	Silver	methylcellulose/sodium hyaluronate/Ag nanoparticle	Suppress M1 microglia activity	[58]
Others	Black phosphorus quantum dots	epigallocatechin-3-gallate@black phosphorus quantum dots	Akt-GSK3	[59]
	Mxene	GelMA-Mxene	Not mentioned	[60]

## Data Availability

No new data were created or analyzed in this study. Data sharing is not applicable to this article.

## Abbreviations

The following abbreviations are used in this manuscript:

CSPGs	chondroitin sulfate proteoglycans
MEP	motor evoked potential
DRG	dorsal root ganglion
VGCCs	voltage-gated calcium channels
NSCs	neural stem cells
PPy	Polypyrrole
PANI	Polyaniline
PEDOT	Poly(3,4-polyvinylpyridine)
CNTs	carbon nanotubes
rGO	reduced graphene oxide
KNN	potassium sodium niobate
